# The African citrus psyllid *Trioza erytreae*: An efficient vector of *Candidatus* Liberibacter asiaticus

**DOI:** 10.3389/fpls.2022.1089762

**Published:** 2022-12-22

**Authors:** Bernard Reynaud, Patrick Turpin, Florencia M. Molinari, Martial Grondin, Solène Roque, Frédéric Chiroleu, Alberto Fereres, Hélène Delatte

**Affiliations:** ^1^ Université de la Réunion, UMR PVBMT, Saint Pierre, Réunion; ^2^ CIRAD, UMR PVBMT, Saint Pierre, Réunion; ^3^ Instituto de Ciencias Agrarias, Consejo Superior de Investigaciones Científicas, ICA-CSIC, Madrid, Spain; ^4^ CIRAD, UMR PVBMT, Antananarivo, Madagascar

**Keywords:** Huanglonbing, *Diaphorina citri*, transmission efficiency, HLB (citrus greening), CLAS

## Abstract

**Introduction:**

Huanglonbing (HLB) is the most serious disease of citrus in the world, associated with three non-cultivable phloem-restricted bacteria *Candidatus* Liberibacter asiaticus (*C*Las), *Ca* L. africanus (*C*Laf) and *Ca* L. americanus (*C*Lam). *C*Las is transmitted by the Asian citrus psyllid *Diaphorina citri*, and has spread to several countries. The African psyllid *Trioza erytreae*, the vector of *C*Laf occurs in Africa and neighbouring islands. Only two major citrus-growing regions - Australia/New Zealand and the Mediterranean Basin - are still HLB-free in the world. However, *T. erytreae* has recently been introduced into continental Europe (Portugal and Spain) and has become a potential threat to citrus production. The transmission of *C*Las by *T. erytreae* had been postulated but never tested. To evaluate the risk of *T. erytreae* transmitting *C*Las, comparative transmissions of *C*Las by *T. erytreae* and *D. citri* were assessed.

**Methods:**

Transmission tests were performed on excised leaves and seedlings of *Citrus volkameriana* with different inoculation access periods (in series) for both insect species. Quantifications of bacterial titers were made in excised leaves, seedlings three and six months after inoculation and on individual insects.

**Results:**

Our results showed that *T. erytreae* was able to efficiently acquire *C*Las. Furthermore, *T. erytreae* carried significantly higher bacterial titers than *D. citri*, and was able to efficiently transmit the bacteria to seedlings at a similar rate that *D. citri* highlighting the high risk of spread of the most aggressive variant of HLB (*C*Las) by *T. erytreae* in Europe.

**Discussion:**

Thus, extreme precautions to prevent any entry of *C*Las into Europe should be adopted.

## Introduction

All Citrus trees belong to the family Rutaceae, subfamily Aurantioideae and are native to the tropical and subtropical regions of Southeast Asia, northeast India, Yunnan Province in southwest China, northern Myanmar, the Indochinese peninsula and the Malaysian archipelago (Zhong and Nicolosi, 2020). It is the world’s largest fruit crop, cultivated in more than 140 countries in the world [143.755, 6 million tons in 2020, (FAO, 2021)]. The main threat of this tree crop is a disease called Huanglongbing (HLB) or citrus greening, which has provoked the decline of many citrus industries that have faced the disease (Gottwald, 2010). The disease was first reported in China in 1919 but the causal agents were only discovered in 1970 by electron microscopy (EM) (Bové, 2006). It is caused by three main species of uncultivable, phloem-restricted, Gram-negative bacteria of the genus *Liberibacter*. Three different bacteria species pathogenic to *Citrus* inducing HLB had been described so far: the Asian species named *Candidatus* Liberibacter asiaticus (*C*Las), the African species *Ca.* Liberibacter africanus (*C*Laf) and the American species *Ca*. Liberibacter americanus (*C*Lam) (Bové, 2006). The former species is described as the most aggressive species among all three causing more severe symptoms of HLB, and being able to grow in a wide range of temperatures, even above 30°C (Bové, 2006). Symptoms affect the entire citrus plant, from roots to leaves, even altering the chemical characteristics and sensory attributes of the fruit, and eventually killing the plant (Bové, 2006; Baldwin et al., 2010; Dala-Paula et al., 2019).

HLB disease is transmitted by grafting and mainly by two species of phloem-sap feeding psyllids (Bové, 2006). One species originating from Asia, the Asian citrus psyllid (ACP), *Diaphorina citri* Kuwayama, 1908 (Hemiptera: Psyllidae) is the vector of *C*Las (Martinez and Wallace, 1967) and *C*Lam (Teixeira et al., 2005a; Texeira et al., 2005b). The second species originating from Africa, the African citrus psyllid (AfCP), *Trioza erytreae* (Del Guercio, 1918) (Hemiptera: Triozidae) (McClean and Oberholzer, 1965) is the vector of *C*Laf (Bové, 2006). Both psyllid species are very sensitive to changes in temperature and relative humidity. The AfCP, develops under cool and moist climate, the optimum temperature for its preimaginal development is between 17°C and 25°C, and its juvenile stages can be completed in 17 and 43 days at 25 and 14°C, respectively (Catling, 1973). The ACP has a higher heat tolerance and its development from egg to adult can be completed in 17 days with optimal population growth potential at 25-28°C (Hall, 2008).

Huanglongbing disease affects several countries in Asia, sub-Saharan Africa, the islands of the Indian Ocean and the Americas (except for Bolivia, Chile, Perú, and Uruguay), but the Mediterranean Basin and Australia are still free from it (Ferrarezi et al., 2020). However, *T. erytreae* has recently been described in southern Europe in Portugal and Spain and is extending its range in both countries eastwards reaching the Basque region just at the border of southern France and southwestwards, reaching the Algarve region in southern of Portugal (Cocuzza et al., 2017; EPPO, 2021; Benhadi-Marín et al., 2022). Most studies on *C*Las transmission have been conducted with *D. citri*, the species widely present in Asia and America, while those on *C*Laf have exclusively used *T. erytreae* as a vector as the pathogen and the vector were previously restricted to the African continent. However, in recent years *C*Las has been introduced into Africa where both psyllid vector species are present and the epidemiological implications of such introduction are unknown (Ajene et al., 2020b). As one of *C*Las’s potential vectors is already present in southern Europe, the high heat tolerance, strong symptoms induction leading to the death of trees, high aggressiveness, and wide distribution of *C*Las, make this disease the main threat to this major citrus-growing area. However, the transmission ability of *C*Las by *T. erytreae* has never been fully proven, as i) when the experiment was carried out (in the 1970s), it was not possible to discriminate HLB species, and ii) it was tested on very few plants (Massonie et al., 1976). Recently, *C*Las was reported from field-collected adults of *T. erytreae* feeding on HLB infected citrus trees in Ethiopia and Uganda (Ajene et al., 2020b), but no transmission assay was conducted to prove its transmission capacity. Thus, there is no information on the risk of spread of *C*Las in a geographical area where *T. erytreae* is present. To clarify the transmission capacity of *C*Las by *T. erytreae*, we compared the transmission capacities of the two psyllids *D. citri* and *T. erytreae* for *C*Las in laboratory experiments. These experiments were conducted in La Réunion, an island in the southwest part of the Indian Ocean where the two-psyllid species are present, and the two HLB species *C*Las and *C*Laf have been reported (Catling, 1972; Garnier et al., 1996; Lu et al., 2021; Wang et al., 2021).

## Material and methods

### Insect material

Laboratory colonies of *D. citri* and *T. erytreae* were started from adult field collected individuals in La Réunion in 2019 and 2020, respectively. These adults were transferred in separate cages and growth chambers to start laboratory colonies for all further experiments in insectarium facilities of the Plant Protection Platform, La Réunion.

*Diaphorina citri* adults were collected on orange jasmine [*Murraya paniculata* (L.) Jack] at 400 m *asl* in Le Tampon locality in La Réunion. The colony was maintained on the same plant with fluctuating temperatures (26°C day/20°C night (+/- 2°C); LD 12:12h photoperiod; 80+/-20% relative humidity).

*Trioza erytreae* adults were initially collected on Tangor Ortanic Mandarin at 700 m *asl* in Salazie locality in La Réunion after an intensive survey, as this species was considered almost extinct in the 90s (Aubert, 1987; Aubert et al., 1996). The colony was maintained on Volkamer lemon (*C. limonia* Osbeck ‘Volkameriana’) with fluctuating temperatures (23°C day/17°C night (+/- 2°C); LD 12:12h photoperiod; 80 +/-20% relative humidity) that should be in the optimal range for development of this species (Cocuzza et al., 2017).

Individuals from the two colonies were qPCR-assayed for *C*Las and *C*Laf absence. The two colonies were regularly checked to ensure their bacteria-free status.

### Plant material and HLB inoculum

In all further laboratory transmission experiments, leaves or seedlings of Volkameriana were used. Those plants were grown in the laboratory growth chamber facilities to ensure their HLB-free status.

*C*Las-infected leaves of plants to be used as *C*Las sources for positive controls and *C*Las acquisition by insects in transmission tests were collected from infected citrus plants grown in a specific orchard at 160 m asl in Saint-Pierre. Two different *C*Las-infected cultivars were used as infected sources for the transmission tests: ForEl 41 (V1), a hybrid between the mandarin Fortune and the Tangor Ellendale and Pet Yala Mandarin (V8) a *Citrus suhuiensis* (Koizumi et al., 1993). The *C*Las-infected status of both infected-source plants was checked prior to the start of the experiments. Both cultivars were chosen because they had different initial bacterial titers (Cq).

### DNA extraction and quantitative CLas molecular detection

Each adult psyllid was individually extracted using the Animal Blood and Tissue kit (Qiagen^©^, Europe) following the manufacturer’s instructions. The DNA was extracted for all psyllids that were used in the experiments, even the collected dead ones that died before the last inoculation access period (IAP).

Each leaf used for the IAP and the acquisition access period (AAP) was individually extracted for its DNA content. To do this, the midrib was separated from each leaf, using a sterile scissor (sterilized after each midrib sampled) directly after sample collection, then stored at -80°C until further use. Then, 0.2 g of each frozen midrib was subjected to a first grinding step in 1mL of Tris-EDTA-SDS buffer (Cellier et al., 2020) using ceramic beads in a FastPrep^®^96 homogenizer (MP Biomedicals, France). This shredded material was then extracted with the DNeasy Plant Mini kit (Qiagen^©^, Europe) following the manufacturer’s instructions. All samples were stored at −80°C.

To determine the *C*Las titer for each extracted individual psyllid or leaf we used a Real time PCR StepOne PLUS (Applied Biosystem^©^, Life Tech) with the following primer sets amplifying a region of the *C*Las 16s rDNA: HLBas TCGAGCGCGTATGCAATACG and HLBr GCGTTATCCCGTAGAAAAAGGTAG and Taqman probe HLBp FAM-AGACGGGTGAGTAACGCG (Li et al., 2006) following a modified protocol of Cellier et al. (2020). Each reaction was made in a final volume of 13 µL comprising: 6.50 μl of GoTaq^®^ Probe Master Mix (Promega ^©^, Europe), 0.25 µM of primers, 0.13 μM of HLBp probe, 3.67μl of water and 2 μl of DNA. The PCR parameters were: 95°C for 10 min, 45 cycles (95°C for 15 sec, 58°C for 1 min). Each sample was run in duplicate and the average Cq value of the two runs was used for assessing *C*Las status. In each qPCR reaction plate, at least 4 negative controls were added (i.e., for plants, DNA samples came from healthy non-*C*Las positive plants, for psyllids it came from DNA samples extracted from non-*C*Las positive insects of each species). The positivity threshold was chosen as the lowest Cq of the controls.

### HLB transmission assays

To evaluate the ability of acquisition, retention and transmission of *C*Las by *T. erytreae* and *D. citri*, we carried out serial inoculation tests.

**Figure 1:**
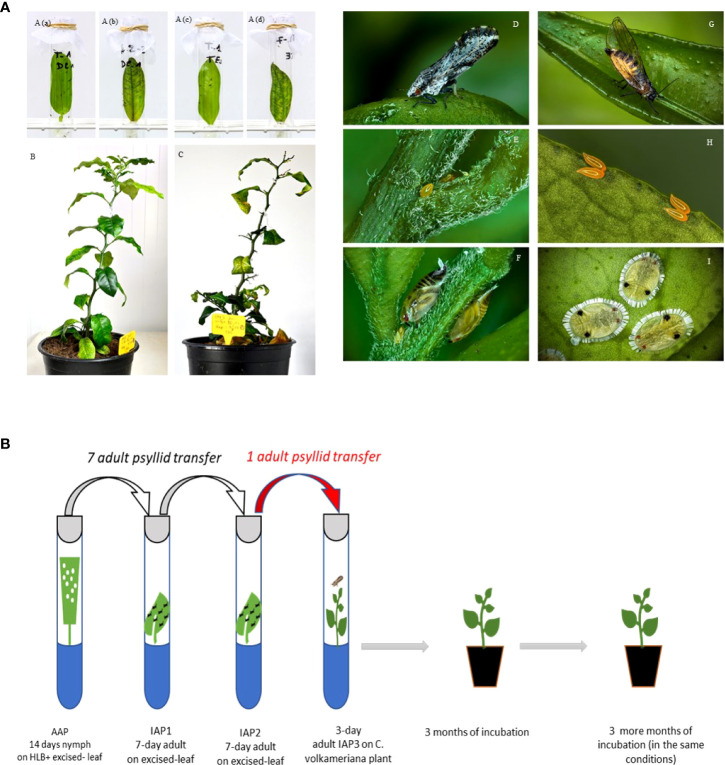
Part **(A)** Different plant material (excised citrus leaves and plants) and insect stages of both psyllid species that were used in the *C*Las transmission experiments. Acquisition access period (AAP) of *Diaphorina citri* **(Aa, Ab)** and *Trioza erytreae* ***(*Ac, Ad*)*** on HLB+ **(Ab, Ad)** or HLB- **(Aa, Ac)** excised leaves*; C*Las + 3-month **(B)** and 6-month **(C)** old plant in IAP 3; *Diaphorina citri* adult **(D)**, eggs **(E)** and nymphs **(F)**; *Trioza erytreae* adult **(G)**, eggs **(H)** and nymphs **(I)**. All pictures were taken by Antoine Franck (CIRAD, UMR PVBMT). Part **(B)** Experimental set up.

The whole experiment consisted in four steps (**Figure 1**). The first step was the AAP where approximately 40 nymphs at the 3^rd^ to 4^th^ instar stage of each species were introduced into tubes containing *C*Las-infected detached leaves from a young field-collected plant (either V1 or V8 mandarin cultivars). In addition, a laboratory-grown detached young leaf of Volkameriana free of *C*Las was used as a negative control. Each petiole was inserted into an Eppendorf tube filled with Murashige and Skoog (MS) medium, itself inserted in a glass tube closed by a fine mesh. The nymphs were left to feed and develop on the infected leaves inside the tubes for an AAP of about 14 days until the adults emerged. As nymphs acquire *C*Las in a much more efficient way than adults, the nymphal stage was chosen for an optimal acquisition (Ammar et al., 2016). After the 14-day AAP, leaves were directly collected and midrib sampled as described above, and frozen at -80°C for further tests.

Then, 5 to 7 newly emerged adults were transferred to a tube containing a healthy Volkameriana detached receptor leaf for the first 7-day of IAP1. Then, the adults (including those developed on non-infected leaves that were used as control) were transferred to another tube containing a healthy Volkameriana detached receptor leaf for the last 7-day of IAP (IAP2). After IAP1 and 2, the excised leaves were left in the tubes for an extra incubation step (in the same thermal conditions) for 7 extra days to allow bacterial multiplication according to Ammar et al. (Ammar et al., 2013). Then, leaves were collected and midrib sampled as described above, and frozen at -80°C for further tests.

To assess the effect of temperature on *C*Las bacterial titer in the leaves and insects during the different AAP and IAP1 and IAP2 tests, we carried out experiments at two different temperature regimes: 23°C day/17°C night or 26°C day/20°C night.

Finally, a further and final IAP (IAP3) was carried out using a single adult from the serial IAP tests which was individually transferred to healthy 2-leaf stage Volkameriana receptor seedlings (young seedlings of 3 to 4 leaves were transferred in tubes on Ms medium) for a 3-day of IAP period. Adults of both species that developed on uninfected leaves were also transferred individually to healthy Volkameriana seedlings under the same conditions as described above and used as controls. After the 3-day of IAP3, all adults were carefully collected and stored individually in absolute ethanol at -80°C for further tests. After IAP3, the seedlings were transferred to 1 L pots in a growth chamber at 26°C+/-2°C (LD 12:12h photoperiod, 80+/- 10% relative humidity) for an incubation period of 3 months. After this incubation period, the 4^th^ upper leaf (from the top) was collected and midrib sampled as described above, and frozen at -80°C for further tests. Plants were then kept for an extra period of 3 months and were checked further for new positives after this period (**Figure 1**).

### Statistical analysis

Analyses were performed using R software version 4.2.0 (R Development Core Team, 2015). To evaluate the effect of temperature, psyllid species, initial *C*Las-infected source cultivars and their interactions, we built linear mixed models on Cq values for AAP, IAP1, IAP2 leaves, IAP3 seedlings and psyllid adults, and generalized linear mixed models on the proportion of positive psyllid adults or IAP3 seedlings (Bates et al., 2015), with the trials as a random effect. Deviance tests were calculated to test the fixed effects. For pairwise comparisons in case of significant fixed effects, we calculated estimated marginal means (Lenth, 2022) and used the Benjamini-Hochberg method for correction of the p-values. Confidence intervals (CI) at 95% were calculated using the functions MeanCI (DescTools package) and/or t.test (R stats package) with conf.level=0.95.

## Results

All field collected leaves of both mandarin cultivars, V1 and V8, were *C*Las positive. All plants or excised leaves where insects from the two species tested *C*Las negative were always found *C*Las negative (negative controls).

**Figure 2:**
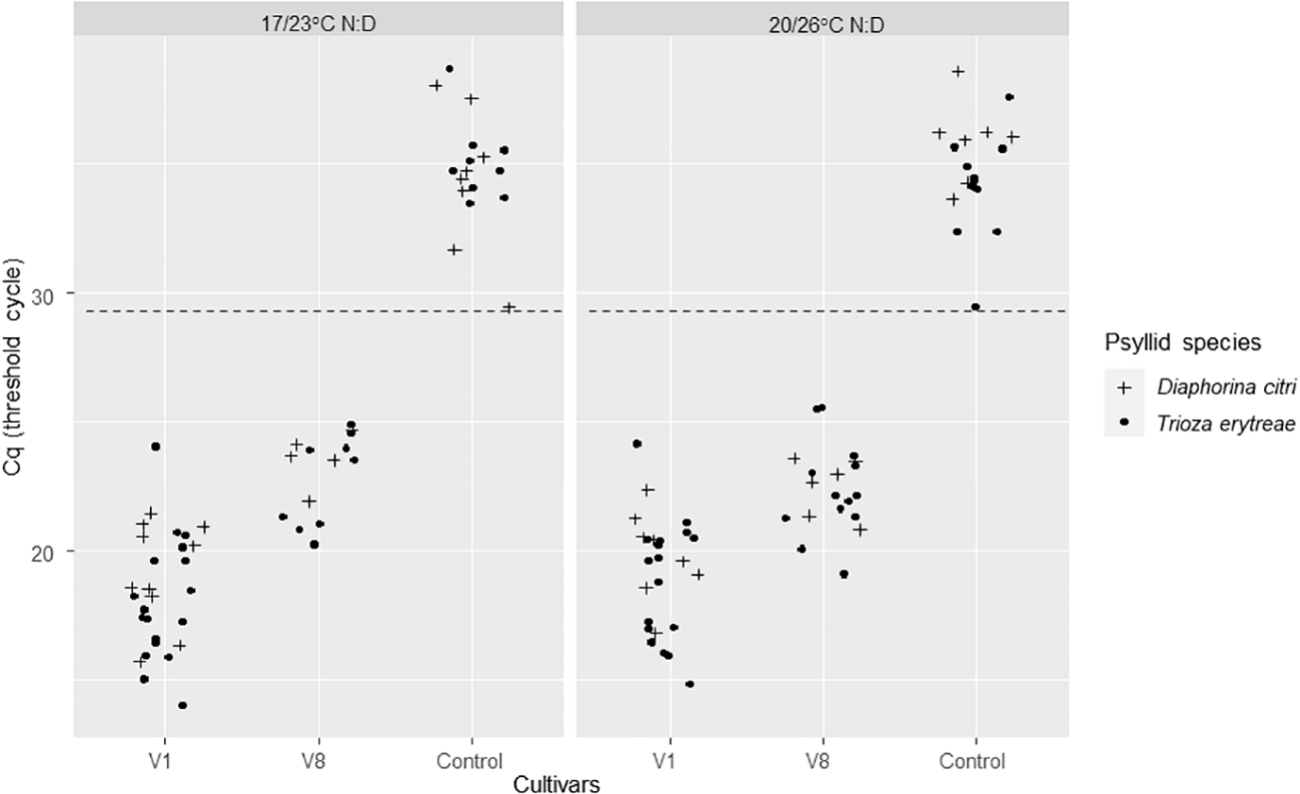
*C*Las Cq values of excised ForEl 41 (V1) and Pet Yala (V8) mandarin cultivars where *Diaphorina citri* and *Trioza erytreae* where in 7-day acquisition access periods (AAP) as nymphs. The control leaves came from a *C*Las – plant of Volkameriana citrus. The positivity threshold (dash line) chosen as the lowest Cq of the controls was of 29.27. AAPs were made at two different sets of temperatures: 17/23°C; night/day or 20/26°C; night/day.

For AAP leaves, the positivity threshold was 29.27. A high significant difference on Cqs between the two *C*Las-infected cultivars was found (Deviance test, P = 1.41 ^-08^) with significantly lower predicted averaged Cq for V1 than V8. There was no significant effect of temperature (Deviance test, P = 0.60) nor between the two psyllid species tested, *T. erytreae* and *D. citri* (Test of deviance, P = 0.22) (**Figure 2**; **Table 1**) on the Cq values.

**Table 1:** Average *C*Las Cq (av. Cq) and numbers of repetitions (n) for each AAP, IAPs, and insects with 95% confidence intervals (CI 95%).

			AAP			IAP1			IAP2			IAP3			Insects		
Psyllid	T°C	Cultivar	n	av.Cq	CI 95%	n *C*las +/Tot	av.Cq	CI 95%	N *C*las +/Tot	av.Cq	CI 95%	N *C*las +/Tot	av Cq	CI 95%	n *C*las +/Tot	av.Cq	CI 95%
DC	17/23	V1	10	19.1	[17.7 - 20.6]	2/12	30.4	[17.6 43.1]	5/10	30.5	[28.1- 33.0]	2/31* (41)**	20.7	[0 - 45.0]	41/54	25.6	[24.5- 26.6]
		V8	5	23.5	[22.3 - 24.8]	0/4	–	–	1/3	31.9		1/6*(19)**	19.3		7/22	27.0	[23.9-30.1]
		Volka	8	34.4	[32.0- 36.7]	0/7	–	–	0/7			0/0* (9)**			0/11		
	20/26	V1	8	19.8	[18.4 - 21.2]	4/6	31.7	[30.5 - 33.0]	2/7	32.2	[26.1 - 38.3]	1/21* (28)**	16.7	–	27/34	26.4	[25.0 27.7]
		V8	6	22.4	[21.2 - 23.6]	0/5	–	–	1/6	29.0		0/10* (19)**		–	13/22	26.9	[25.3 28.5]
		Volka	7	35.8	[34.4 - 37.3]	0/6	–	–	0/7			0/0* (10)**			0/11		
TE	17/23	V1	18	18.0	[16.8 - 19.2]	4/20	32.2	[31.4 - 33.0]	4/18	32.6	[32.2 - 33.0]	3/32* (34)**	19.9	[18.0- 21.8]	47/51	20.3	[19.6-21.0]
		V8	9	22.7	[21.3 - 24.1]	0/9	–	–	1/8	30.6		0/15* (28)**	–	–	21/37	24.4	[23.2- 25.6]
		Volka	9	35.1	[33.9 - 36.3]	0/6	–	–	0/7			0/0* (11)**			0/11		
	20/26	V1	17	18.8	[17.5 - 20.1]	1/13	31.0	–	5/14	30.9	[29.1- 32.8]	2/26* (28)**	17.6	[2.27-32.9]	32/35	18.9	[18.0-19.8]
		V8	26	22.3	[21.2 - 23.5]	0/11	–	–	0/6			1/16* (16)**	18.2	–	16/16	20.8	[19.0- 22.6]
		Volka	11	34.1	[32.6 - 35.5]	0/8	–	–	0/8			0/0* (6)**			0/9		

This data is presented according to acquisition on cultivars V1 (ForEl 41) or V8 (Pet Yala) mandarin or Volka (Volkameriana), and different temperature sets (T°C) for each psyllid species *Diaphorina citri* (DE) and *Trioza erytreae* (TE). For IAP 1 and 2, batches of 5 to 7 insects were used to inoculate excised leaves, whereas for IAP 3, individual insects were transferred from insect batches of IAP2 on single seedlings.

*number of plants from positive insects; **total number of plants.

**Figure 3:**
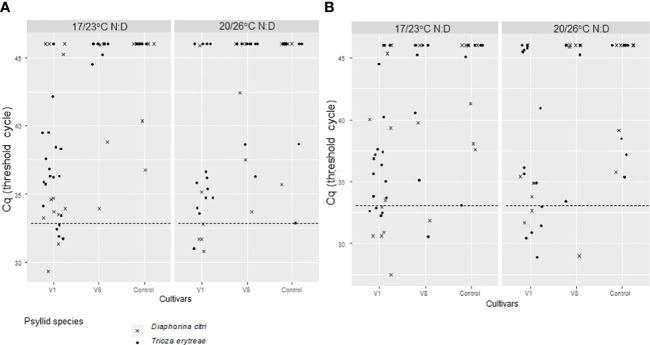
*C*Las Cq of excised Volkameriana citrus leaves where *Diaphorina citri* and *Trioza erytreae* where in 7-day inoculation access periods IAP1 **(A)** and IAP2 **(B)** as adults. The 7-insect batches that were previously on AAP on *C*Las + V1 (ForEl 41) or V8 (Pet Yala) mandarin cultivars and *C*Las- Volkameriana (**Figure 2**) where transferred on new Volkameriana *C*Las – excised leaved for two 7-day IAPs and tested by qPCR for *C*Las detection. The positivity threshold (dash line) chosen as the lowest Cq of the controls was of 32.9 for IAP1 and 33.1 for IAP2. IAPs were made at two different sets of temperatures: 17/23°C; night/day or 20/26°C; night/day.

For IAP1 excised leaves, the positivity threshold was 32.86. A significant difference between psyllid species (Deviance test, P = 0.02) and between cultivars on Cq values was found (Deviance test, P = 0.03) (**Figure 3A**; **Table 1**). In IAP1, 22.22% (n=27) of the excised leaves were tested *C*Las positive by *D. citri*, and 9.43% (n=53) by *T. erytreae* after the 7-day IAP. When looking at the initial *C*Las-infected sources used for acquisition (AAP) (V1 and V8 cultivars), all IAP1 positive excised leaves (for both psyllid species) were derived from the V1 cultivar (**Figure 3A**; **Table 1**).

For IAP2 excised leaves, the Cq threshold was 33.12. No significant effect of temperature, initial *C*Las-infected source cultivar, or psyllid species was observed on the number of positive psyllids (Deviance test, P > 0.09). We observed 34.62% (n=26) of the excised leaves that were *C*Las positive when inoculated by *D. citri*, and 33.33% (n=45) when inoculated by *T. erytreae* after the last 7-day IAP on observed data (**Figure 3B**; **Table 1**). When predicted values were calculated by emmeans (*D. citri* predicted values= 23% [1-89%, 95% CI]; *T. erytreae* predicted values=4% [0-51%, 95% CI]), strong discrepancies were observed with the observed values, most probably due to too small samples. When looking at the initial *C*Las-infected source plants leaves used for acquisition (AAP) (both V1, V8), 66.67% (n=9) and 90% (n=10) IAP2 positive excised leaves were derived from V1 cultivar for *D. citri* and *T. erytreae*, respectively (**Figure 3B**; **Table 1**).

**Figure 4:**
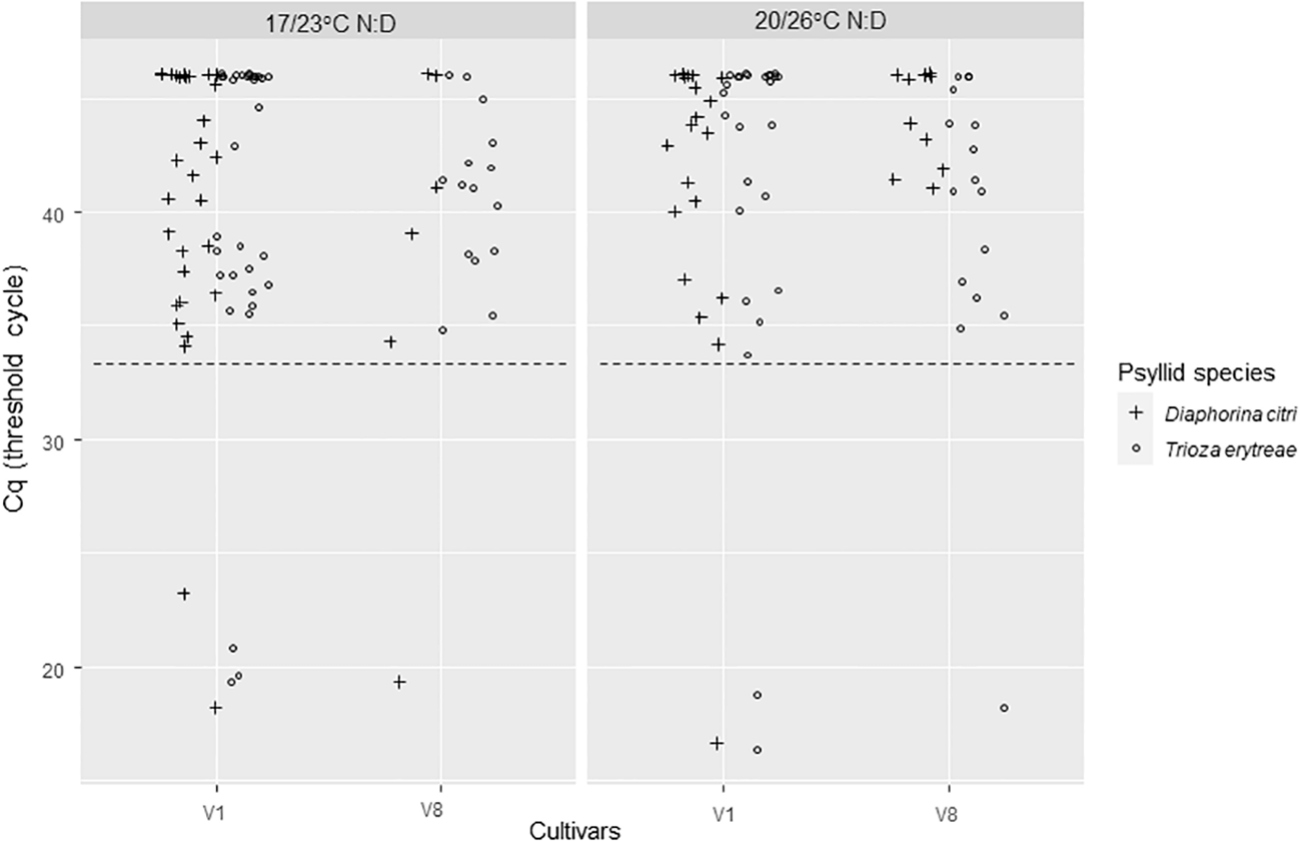
*C*Las Cq of 3-month Volkameriana citrus plants where *Diaphorina citri* and *Trioza erytreae* where in 3-day inoculation access periods at the IAP3. One adult insect that were previously on IAP2 (**Figure 3**) was transferred on a Volkameriana *C*Las – seedlings for a 3-day IAP. Plants where further left to grow in growth chambers in the lab for 3-month, then tested by qPCR for *C*Las detection. The positivity threshold (dash line) chosen as the lowest Cq of the controls was of 33.28. IAPs were made at two different sets of temperatures: 17/23°C; night/day or 20/26°C; night/day.

For IAP3 seedlings, the positivity threshold was 33.28. No significant effect of temperature, initial *C*Las-infected source cultivar, or psyllid species was observed (Deviance tests, P > 0.51). In total, the predicted transmission rates on 3-month old plants, using one insect by plant, were 7% [1-26%, 95% CI] for *D. citri* and 9% [3-29%, 95% CI] for *T. erytreae* (**Figure 4**; **Table 1**) with averaged Cq from 17 to 22 (**Table 1**). No more positive plants were observed after three more months (i.e., 6-month old plants).

**Figure 5:**
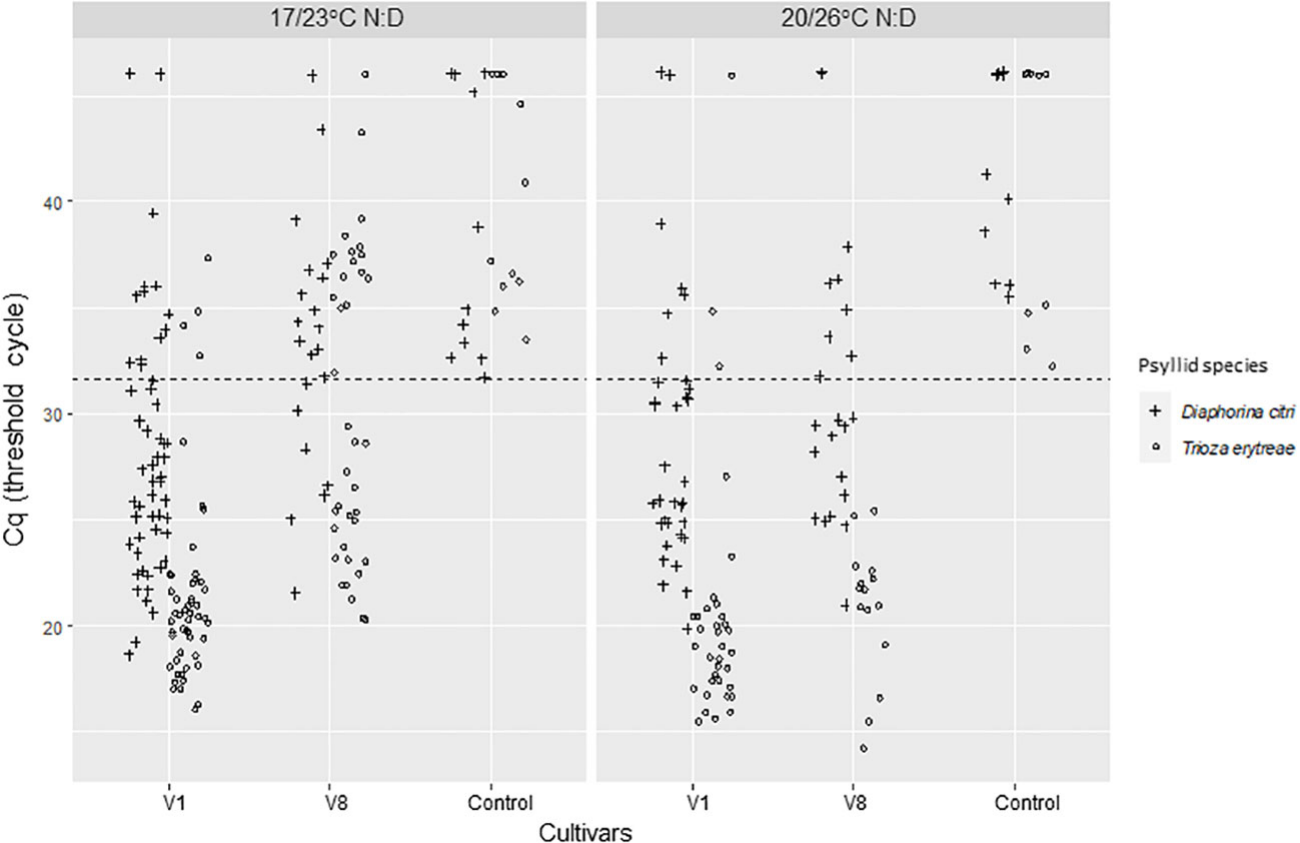
*C*Las Cq of *Diaphorina citri* and *Trioza erytreae* that where used in the whole experiment and collected after the last 3-day IAP3. The positivity threshold (dash line) chosen as the lowest Cq of the controls was of 31.66. IAPs were made at two different sets of temperatures: 17/23°C; night/day or 20/26°C; night/day.

The *C*Las infection status and bacterial titer for all adult psyllids that were used in the experiment (except the ones that were lost) were assessed after the IAP 3 (**Figure 5**). The positivity threshold for this experiment was 31.66. Significant differences were found in the proportion of *C*Las positive psyllid adults between the two species (Deviance test, P = 0.003) with 78% [63-88%, 95% CI] of *D. citri* adults and 92% [80-97%, 95% CI] of *T. erytreae* adults that were *C*Las positive. There was an effect of the initial *C*Las-infected source cultivars used for acquisition (Deviance test, P = 1.28e^-5^). No temperature effect was noticed in the proportion of positive psyllid adults. However, when taking into account Cq values, significant interactions were found between the initial cultivars AAP cultivars and psyllid species (Test of deviance, P= 0.025) as well as for temperature ranges and psyllid species (Test of deviance; P=0.04) (**Figure 5**; **Supplementary Data Figure 1**). These significant interactions were mostly due to *T. erytreae* samples that had much higher Cq values when cultivar V8 was used as a *C*Las-infected source for the AAP at the lowest temperature range (**Supplementary Data Figure 1**). Higher differences were found for *T. erytreae*, with lower averaged Cq values for V1 (20.18 [19.07-21.30, 95% CI] and of 18.57 [17.32-19.83, 95% CI] at 23°C and 26°C, respectively) than for V8 (24.24 [22.79-25.68, 95% CI] and of 20.89 [19.23-22.55, 95% CI] at 23°C and 26°C, respectively). For *D. citri*, higher Cq values were observed on average compared to *T. erytreae*, but with fewer differences between V1 (25.37 [24.23-26.51, 95% CI] and 26.14 [24.87-27.42, 95% CI] at 23°C and 26°C, respectively) and V8 (27.15 [24.88-29.41, 95% CI] and 26.44 [24.73-28.15, 95% CI] at 23°C and 26°C, respectively) were observed (**Supplementary Data Figure 1**).

## Discussion

Our results demonstrated the ability of *T. erytreae* to acquire and transmit *C*Las equally well as *D. citri*, with no effect of the initial bacterial inoculum or temperature ranges tested.

When testing the bacterial titer of different mandarin cultivars as *C*Las-infected source plants for the initial inoculum (AAP) we were able to choose two cultivars (V1 and V8) that were found to have significantly different bacterial titers, both showing strong symptoms in the field. The highest bacterial titer was observed for V1, a cultivar known to be HLB susceptible (Koizumi et al., 1993). These differences in bacterial titers between cultivars had already been shown in the field and could reflect the capacity of the V8 cultivar to diminish bacterial multiplication (Alves et al., 2021). Another hypothesis to explain those observed variations could be that a different variant or strain of *C*Las had infected V1 and V8 cultivars, even in trees next to each other in a same orchard. Genetic studies of the *C*Las species revealed that even within a given region, it might comprise several different variants (Bastianel et al., 2005; Teixeira et al., 2008).

To our knowledge, this is the first time that plant-to-plant transmission experiments using *C*Las-infected plants and *T. erytreae* as the vector species have been performed. Both excised leaves and seedlings were used for both acquisition and inoculation tests. Our results clearly show the efficacy of *T. erytreae* to acquire and transmit efficiently the bacteria (i.e., in a similar way as *D. citri* its primary vector) highlighting the threat of having this vector invading the commercial citrus growing areas of southern Europe (Cocuzza et al., 2017; Benhadi-Marín et al., 2022). More importantly, our results highlight that the most aggressive and destructive *C.* Liberibacter species (*C*Las) can be spread efficiently by *T. erytreae* in regions where *D. citri* has not yet been established. Our findings have important epidemiological implications as *C*Las in addition to *C*Laf can be efficiently spread in regions where *T. erytreae* is present. This stresses the importance of putting in place extreme precautions to prevent the introduction of *C*Las-infected material in countries where *T. erytreae* is already established such as Spain or Portugal that have a very important citrus industry.

The initial AAP inoculum bacterial titer was shown to be important since most of the effective transmissions to the final IAP by the two-psyllid species on seedlings came from the V1 cultivar with the highest initial bacterial titer. A 14-day AAP using nymphs should have ensured a better acquisition rate for insects (Inoue et al., 2009; Ammar et al., 2016), which was verified here with *C*Las-positive rates obtained for *D. citri* and *T. erytreae* of 67 and 83%, respectively. Nonetheless, despite such a positive number of *C*Las-positive insects, only 7% and 9% of transmissions were recorded on IAP3 seedlings for single insect transmission of *T. erytreae* and *D. citri*, respectively. Similar results were observed with single insect transmission of *D. citri* (with transmission rates of 6.7% after 31 days post AAP) showing intermittent and random transmission over series of IAP (Canale et al., 2017). To be noticed, recent results obtained for *D. citri* with *C*Las on citrus seedlings with young shoots gave much higher transmission rates (over 56% of transmission), however this could be possibly explained by the experimental design, slightly different (regarding to AAP and IAP times) and the citrus cultivar used as the recipient of *C*Las (Lopes and Cifuentes-Arenas, 2021). Indeed, Volkameriana cultivar was considered as a moderately tolerant cultivar to *C*Las compared to sensitive sweet oranges, but still with comparable high bacterial titers (Folimonova et al., 2009). Furthermore, different bacterial strains or psyllid populations between both studies could also play a role in these discrepancies, as in La Réunion the *C*Las strain is slightly different from others (Lu et al., 2021) such as the ACP psyllid populations which were found genetically different from the new invasive populations in Kenya (Wang et al., 2021) as being present on the island for over 50 years (Bové, 2006).

*T. erytreae* had significantly higher bacterial titers than *D. citri* (**Supplementary Data Figure 1**), but with no higher transmission rates on seedlings in IAP3, or on excised leaves in IAP1 and IAP2, compared to *D. citri*. *Trioza erytreae* had been shown to acquire (1 hour AAP was enough) and efficiently transmit *C*Laf within less than 7 days post AAP, quicker than *D. citri* with *C*Las (van Vuuren and van der Merwe, 1992; Ammar et al., 2013; Ammar et al., 2016). So, these lesser transmission rates observed for *T. erytreae* with *C*Las (compared to *C*Laf) might reflect a lesser adaptation of *T. erytreae* to *C*Las, as *C*Las never co-evolved due to their non-overlapping geographical distribution until recently (case of Ethiopia and Kenya where both *C*Las and *T. erytreae* are together (Ajene et al., 2020a; Ajene et al., 2020b)). This geographical isolation also contributes to explaining why all transmission tests with *C*Las have been conducted exclusively with *D. citri*, and all the *C*Laf transmission tests have been conducted with *T. erytreae.* Our results showing the almost equal transmission ability of *C*Las by *T. erytreae* need however to be further refined and further experiments should be conducted to better determine the latency period, the persistence or the intermittence of transmission over the life span of *T. erytreae.*

According to our results *T. erytreae* has the capacity of acquisition of *C*Las bacteria at its larval stage and ability to transmit it during its feeding at the adult stage to a *C*Las-free plant. This implies that *C*Las, known to be transmitted in a propagative-circulative manner by *D. citri* (Inoue et al., 2009; Ammar et al., 2016; Canale et al., 2017; Ammar et al., 2018), might also have the same capacity to multiply and circulate up to the salivary glands of *T. erytreae*, a psyllid of a different area of origin and from another family. Nonetheless, further experiments are needed to determine the pathways of passage and multiplication of the bacteria in the insect body and to be able to compare them with the system of *D. citri* and *C*Las.

No significant effect was observed between both temperature regimes tested (17-23°C/20-26°C) on transmission rates of both psyllid species on excised leaves or seedlings. Similar results were observed in the USA on *C*Las-infected plants incubated at 20°C, 27°C, and 32°C where similar bacterial titers and symptom induction were obtained (Folimonova et al., 2009). Those tested temperature ranges are also compatible with the ones where *T. erytreae* is already present in Southern Europe (Cocuzza et al., 2017; Benhadi-Marín et al., 2022). This area is then at high risk if the bacteria is introduced, consequences would be tremendously important knowing that this region, still HLB-free, is the 5^th^ worldwide citrus producer, after China, the USA, Brazil, and India (FAO, 2021).Furthermore, a recent study has proven also the risk of the potential introduction of this vector in high citrus growing areas of Mexico that would be most suitable for this vector than *D. citri* with *C*Las already present (Espinosa-Zaragoza et al., 2021). Preventive measures and an action plans were proposed in previous studies to reduce the risk of introduction and/or establishment of *C*Las and *C*Laf in the Mediterranean area (Durán-Vila et al., 2014; Aragón et al., 2022). Thus, in the light of the results of our study and the recent increase in the range of *T. erytreae*, these proposed measures should be taken into account even more. In conclusion, this study clearly demonstrated the ability of *T. erytreae* to acquire and transmit *C*Las. The presence of *T. erytreae* in the southern Europe is of great concern given its ability to disseminate the most aggressive variant of HLB (*C*Las). Thus, extreme precautions to prevent any entry of *C*Las into Europe should be adopted.

## Data availability statement

The original contributions presented in the study are included in the article/**Supplementary Material**. Further inquiries can be directed to the corresponding author.

## Author contributions

Conceptualization, BR, HD; Data collection, BR, MG, PT, FM, SR; Data analysis and interpretation, PT, FC, BR, HD; Writing—original draft, HD; Writing, review and editing, BR, HD, AF, FC; Funding acquisition, BR, HD, AF. All authors contributed to the article and approved the submitted version.
