# Supplementary material for: The African citrus psyllid Trioza erytreae: An efficient vector of Candidatus Liberibacter asiaticus
Source: Front Plant Sci. 2022 Dec 22;13:1089762. doi: 10.3389/fpls.2022.1089762 (PMC9815554; doi:10.3389/fpls.2022.1089762)
Supplement: Supplementary file 1 [file Image_1.pdf]

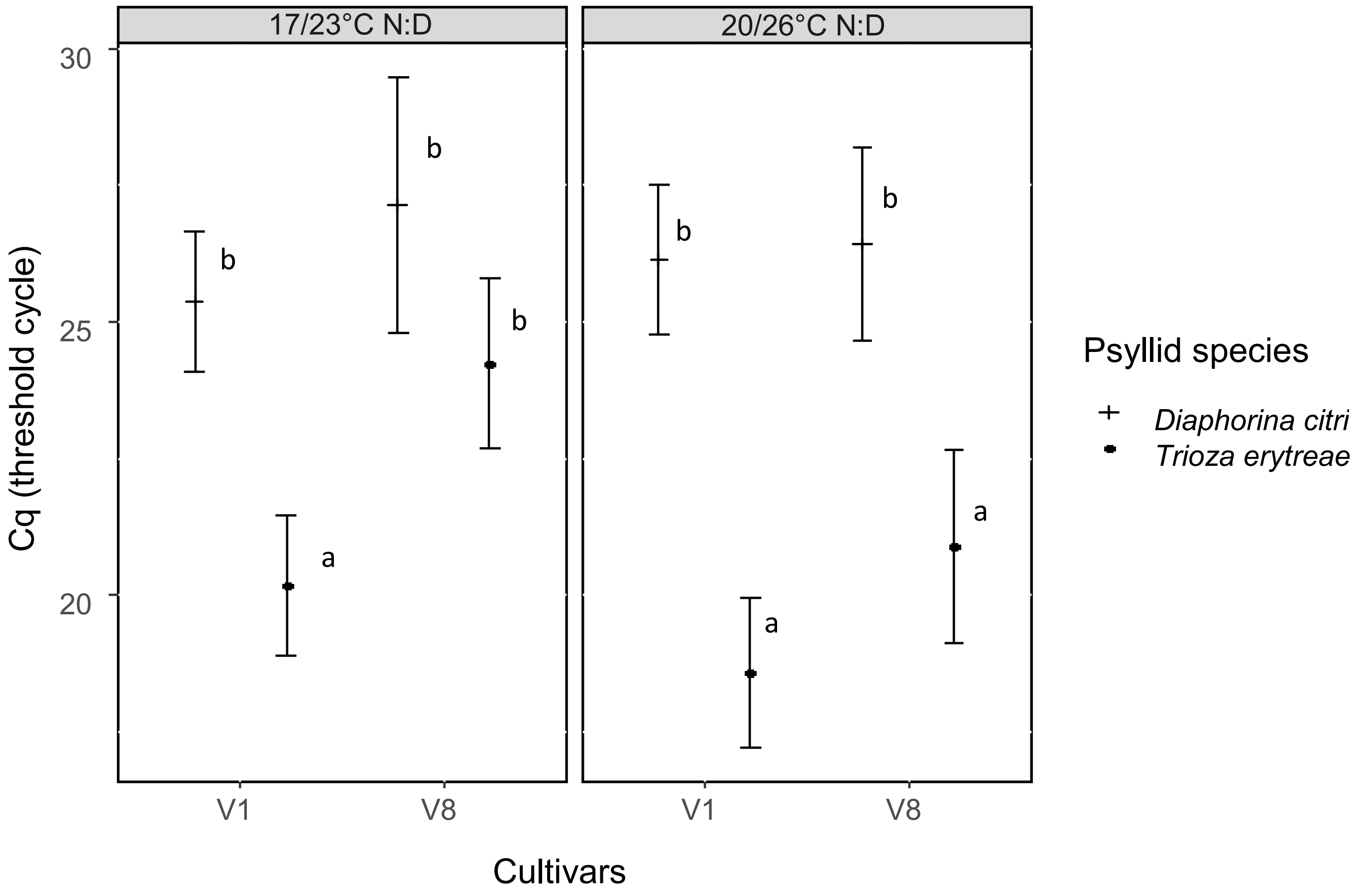

Supp data figure 1. Average predicted values of the *C*Las Cq of *Diaphorina citri* and *Trioza erytreae* that where used in the whole experiment and collected after the last 3-day IAP3.
